# PPARγ as a predictive biomarker for antidepressant response in major depressive disorder: Insights from TNIP1 transcriptional regulation

**DOI:** 10.1016/j.bbih.2025.101151

**Published:** 2025-12-01

**Authors:** Yu-Wei Lin, Daw-Yang Hwang, Yi-Yung Hung

**Affiliations:** a Department of Psychiatry, Kaohsiung Chang Gung Memorial Hospital and Chang Gung University College of Medicine, Kaohsiung, Taiwan; b National Institute of Cancer Research, National Health Research Institutes, Tainan, Taiwan; c Department of Psychiatry, Kaohsiung Municipal Fong Shan Hospital - Under the Management of Chang Gung Medical Foundation, Kaohsiung, Taiwan

## Abstract

**Introduction:**

Major depressive disorder (MDD) is a debilitating disease possibly linked to immune defense mechanisms. TNIP1, a key anti-inflammatory regulator in NF-κB and TLR pathways, is implicated in MDD, but its transcriptional regulation and role in treatment response are unclear.

**Methods:**

We analyzed mRNA expression of TNIP1 and 13 transcription factors in monocytes from pre- and post-treatment (4–6 weeks) MDD patients and healthy controls. Peripheral blood mononuclear cells were isolated, tagged by CD14, and mRNA was extracted and analyzed. Participants were recruited at Kaohsiung Chang Gung Memorial Hospital (2014–2025). ANCOVA, paired t-tests, and multiple linear regression adjusted for age, gender, BMI, and smoking were used to compare groups and predict treatment outcomes.

**Results:**

This study encompassed 62 MDD patients and 52 healthy controls. Pre-treatment HAMD-17 averaged 23.90 ± 4.60, and post-treatment HAMD-17 averaged 8.43 ± 4.24. MDD patients showed higher PPAR-γ (*p* < 0.001), FOS (*p* = 0.023), and lower JUN (*p* = 0.029) expression than controls. Post-treatment, TNIP1 expression increased (*p* = 0.031). Lower pre-treatment PPAR-γ predicted greater symptom improvement (*p* = 0.016).

**Conclusion:**

This study highlights the differential expression of PPAR-γ, FOS, and JUN in MDD patients, underscoring their potential roles in immune regulation. The association between lower pre-treatment PPAR-γ expression and improved treatment outcomes suggests its utility as a biomarker for predicting therapeutic response.

## Highlights

• Lower pre-treatment PPARγ mRNA in monocytes predicts greater improvement in MDD depressive symptoms, indicating its potential as a biomarker.
• MDD patients show significantly higher PPARγ and FOS, and lower JUN mRNA expression compared to healthy controls, reflecting inflammatory mechanisms.
• TNIP1 mRNA expression significantly increases in MDD patients' monocytes after 4–6 weeks of antidepressant treatment.

## 1 Introduction

Major depressive disorder (MDD) is a heterogeneous condition characterized by a variety of symptoms that can lead to cognitive impairments, emotional and psychological symptoms, or manifest as somatic symptoms such as pain and fatigue (Marx et al., 2023). Regarding the inflammatory nature of the mechanisms underlying depression, previous studies have suggested that depressive responses may originate from evolutionarily immune defense mechanisms, which have transformed into pathological inflammation in the context of modern environments (Miller and Raison, 2016). Research has indicated that Interleukin-1β (IL-1β), Interleukin 6 (IL-6), and tumor necrosis factor α (TNF-α) play critical roles in the development of depressive symptoms and motivation systems by influencing neuroplasticity, monoaminergic neurotransmission, and the hypothalamic-pituitary-adrenal (HPA) axis (Bekhbat et al., 2022, 2025; Felger and Lotrich, 2013).

The dynamic activation of the immune system is regulated by post-translational modifications (Liu et al., 2016), which modulate the localization, activity, and abundance of signaling molecules. Deubiquitination, in particular, plays a critical role in suppressing immune responses. The tumor necrosis factor, alpha-induced protein 3 (TNFAIP3) - TNFAIP3 Interacting Protein 1 (TNIP1) complex is frequently highlighted in the context of MDD (Chen et al., 2017; Hung et al., 2017, 2025). TNFAIP3 functions as a biomarker for response to antidepressant treatment, whereas TNIP1 represents the sole TNFAIP3-interacting protein exhibiting selective upregulation in expression among patients with major MDD who achieved remission following chronic antidepressant therapy. Additionally, the lysine-63 deubiquitinase cylindromatosis (CYLD) acts as a negative regulator of inflammation in the Toll-Like Receptor (TLR) signaling pathway, and its activity has been linked to reduced anxiety and depressive-like behaviors (Zajicek et al., 2022). These findings suggest that targeting intrinsic negative regulatory pathways of inflammation could be a promising approach for treating MDD.

In vitro, in the nuclear factor kappa-light-chain-enhancer of activated B (NF-κB) signaling pathway, TNIP1 interacts with TNFAIP3 and the inhibitor of kappaB kinase (IKK) complex to inhibit the nuclear translocation of p65. Within the toll-like receptor (TLR) pathway, TNIP1 suppresses the activation of myeloid differentiation primary response 88 (MyD88) (Zhang et al., 2018) and interleukin-1 receptor-associated kinase 1 (IRAK1) (Nanda et al., 2016), thereby reducing the expression of IL-6 and TNF-α. Furthermore, TNIP1 mitigates inflammation by inhibiting the activation of p38 and c-Jun N-terminal kinase (JNK) in epithelial cells and macrophages, thereby modulating the MAPK signaling pathway (Shamilov et al., 2020). In vivo, animal model showed decreased expression of TNFAIP3-interacting protein 1 (TNIP1) was accompanied by elevated levels of IL-1β, IL-6, and TNF-α in both the serum and colon (Bao et al., 2022), indicating that TNIP1 plays a critical role in attenuating inflammatory responses.

Essentially, TNIP1 plays a crucial role in suppressing inflammation and NF-κB activity, and its transcription is tightly controlled by several regulatory factors, including PPARγ, NF-κB, AP-1, ATF-2, c-Fos, c-Jun, and p53. Among these, PPAR-γ enhances TNIP1 expression through direct promoter binding, thereby reinforcing its anti-inflammatory function (Gurevich et al., 2012b). Activation of PPAR-γ by specific agonists has also been linked to antidepressant effects, particularly in cases of inflammation-associated depression (Sepanjnia et al., 2012).

As NF-κB serves as a positive regulator of TNIP1 (Gurevich et al., 2012a), its activation may initially drive TNIP1 expression. However, chronic NF-κB hyperactivation, a hallmark of MDD (Felger and Lotrich, 2013), promotes inflammation and neurotoxicity. Thus, the antidepressant-associated increase in TNIP1 may represent a counter-regulatory adaptation that mitigates NF-κB–mediated inflammatory signaling. Within the AP-1 transcription factor family (including FOS, JUN, JUNB, and JUND), FOS expression is markedly reduced in stress-induced depressive mouse models, correlating with depression-like behaviors under chronic social defeat stress (CSDS) (Covington et al., 2010). Consistently, downregulation of FOS/JUN has been observed in keratinocytes exhibiting elevated TNIP1 expression (Rudraiah et al., 2018), suggesting potential cross-regulatory mechanisms. ATF2 and ATF3 also modulate stress and inflammatory pathways. Notably, ATF3 expression is altered under chronic stress conditions, implicating it in central stress adaptation and depressive phenotypes (Hai et al., 2010). Furthermore, dysregulation of vitamin D/VDR signaling contributes to neuroinflammation and impaired neuroplasticity in MDD (He et al., 2020), while certain VDR genetic variants have been associated with an increased susceptibility to depression (Lye et al., 2021).

To investigate the potential transcriptional mechanisms underlying the regulation of TNIP1 by antidepressants in depression, this study examines the mRNA expression profiles of TNIP1-related transcription factors in monocytes from MDD patients and healthy controls, both before and after antidepressant treatment. These findings may provide insights into the molecular interplay between inflammation, TNIP1 regulation, and treatment responsiveness in MDD.

## 2 Materials and methods

### 2.1 Experimental design

From August 2014 to Aug 2025, patients hospitalized with major MDD were recruited at Kaohsiung Chang Gung Memorial Hospital. Eligible patients, aged 20–65 years, were diagnosed with MDD based on the criteria of the Diagnostic and Statistical Manual of Mental Disorders, Fifth Edition (DSM-5). The severity of depression was evaluated using the 17-item Hamilton Depression Rating Scale (Ham-D). Blood samples were collected after excluding patients with systemic diseases, such as cardiovascular, liver, or thyroid diseases, or those with alcohol dependence. Following the initial blood draw, antidepressants were prescribed based on the clinicians' judgment of the best treatment for each patient. Four to six weeks later, a second blood sample was obtained. Healthy controls were recruited after semi-structured interviews confirmed the absence of psychiatric disorders according to DSM-5 criteria. All participants provided written informed consent after the study's purpose and procedures were fully explained. The Institutional Review Board (IRB) of Chang Gung Memorial Hospital approved the study design (103–6984A3, 103–5114B, 104–9562A3, 201700153B0, 201700539A3, 202102155B0).

### 2.2 Monocyte isolation, mRNA extraction, and quantitative reverse transcription-polymerase chain reaction (qRT-PCR) analysis

Venous blood samples (5 mL) were collected, and peripheral blood mononuclear cells (PBMCs) were isolated using methods adapted from prior research (Hung et al., 2017). PBMCs were separated from the blood and tagged with anti-human CD14 magnetic beads. The CD14(+) monocyte fraction was subsequently isolated for downstream analysis. Total RNA was extracted from either purified monocytes using the Quick-RNA MiniPrep Kit (Zymo Research, Orange, CA, USA). RNA purity was assessed with a NanoDrop spectrophotometer, ensuring an OD260/OD280 ratio of 1.8–2.0. Immediately after processing, samples were stored at −80 °C until analysis. The mRNA analysis protocol, including primer sequences, followed established methods (Chiang et al., 2021).

### 2.3 Quantitative reverse transcription-polymerase chain reaction analysis

Triplicate quantitative reverse transcription-polymerase chain reaction (qRT-PCR) was performed using the following sets of primers:

**Table:** 

Gene Name	Forward Primer (5' → 3′)	Reverse Primer (5' → 3′)
PPAR-γ	ACCAAAGTGCAATCAAAGTGGA	ATGAGGGAGTTGGAAGGCTCT
PPAR-δ	ACTGAGTTCGCCAAGAGCATC	ACGCCATACTTGAGAAGGGTAA
AR	TCACCGCACCTGATGTGTG	ACATGGTCCCTGGCAGTCTC
ESR1	CAGGAACCAGGGAAAATGTG	AACCGAGATGATGTAGCCAGC
ESR2	ACTTGCTGAACGCCGTGACC	CAGATGTTCCATGCCCTTGTT
PGR	TGAATCCGGCCTCAGGTAGTT	CGCGCTCTACCCTGCACTC
NR3C1	GAGGGAAGGAAACTCCAGCC	TCAGCTAACATCTCGGGGAA
NR3C1	GAGGGAAGGAAACTCCAGCC	GCGCCAAGATTGTTGGGATG
VDR	TTTGGGTCTGAAGTGTCTGTG	GTTCCGGTCAAAGTCTCCAG
NOTCH1	GTCAACGCCGTAGATGACC	TTGTTAGCCCCGTTCTTCAG
NR2F1	ATCGTGCTGTTCACGTCAGAC	TGGCTCCTCACGTACTCCTC
ATF2	AATTGAGGAGCCTTCTGTTGTAG	CATCACTGGTAGTAGACTCTGGG
ATF3	CCTCTGCGCTGGAATCAGTC	TTCTTTCTCGTCGCCTCTTTTT
FOS	CTGGCGTTGTGAAGACCAT	TCCCTTCGGATTCTCCTTTT
JUN	ATCAAGGCGGAGAGGAAGCG	TGAGCATGTTGGCCGTGGAC
JUNB	ACAAACTCCTGAAACCGAGCC	CGAGCCCTGACCAGAAAAGTA
JUND	TCATCATCCAGTCCAACGGG	TTCTGCTTGTGTAAATCCTCCAG
TP53	CAGCACATGACGGAGGTTGT	TCATCCAAATACTCCACACGC

The relative abundance of mRNAs was calculated based on the threshold cycle (CT), where the difference in CT (-ΔCt) used to represent relative expression of clinical samples was defined as CT_GAPDH_ - CT_sample_.

### 2.4 Statistical analysis

All results are reported as mean ± standard deviation. Demographic differences, such as sex, were analyzed using the Chi-square test. Differences in age and body mass index (BMI) between groups were assessed with Student's t-test. Group comparisons were conducted using Analysis of Covariance (ANCOVA), adjusting for age, sex, BMI, and smoking status. Paired t-tests were used to compare mRNA levels in patients with MDD before and after treatment. Multiple linear regression was employed to examine the association between pretreatment mRNA levels and changes in HAMD-17 scores post-treatment. In addition, Receiver Operating Characteristic (ROC) analysis was performed to evaluate whether different transcription factor levels could distinguish between responders and non-responders. Data analysis was performed using SPSS 19 (Chicago, IL, U.S.A.). *p*-values of <0.05 were considered statistically significant.

## 3 Results

### 3.1 Demographic and clinical characteristic

This study included 114 participants, of which 52 were healthy controls and 62 were cases of MDD. The MDD group had a mean age of 46.14 ± 10.35 years, significantly older than the healthy controls at 39.44 ± 10.34 years (*p* = 0.001). Gender distribution showed 16 males and 46 females in the MDD group, and 10 males and 42 females in the control group, with no significant difference (*p* = 0.405). The MDD group had a higher mean BMI of 24.82 ± 4.45 kg/m2 compared to 23.04 ± 3.64 kg/m2 in controls (*p* = 0.023). Smoking was significantly more prevalent in the MDD group (29 smokers, 33 non-smokers) than in controls (3 smokers, 49 non-smokers; *p* < 0.001). In the MDD group, the pre-treatment Hamilton Depression Rating Scale (HAMD-17) score averaged 23.90 ± 4.60, and the averaged post-treatment HAMD-17 was 8.43 ± 4.24. In the population with MDD, the types of medications used are as follows: SSRIs accounted for 21 % (13 out of 62), SNRIs accounted for 50 % (31 out of 62), and other types accounted for 29 % (18 out of 62). Detailed demographic data are provided in Table 1, which showed significant difference in age, BMI, and smoking between these two groups.

**Table 1:** Demographic and clinical data on healthy controls and MDD patients.

	MDD (n = 62)	Health control (n = 52)	*p*-value
Age (years)	46.14 ± 10.35	39.44 ± 10.34	0.001∗
Gender (M/F)	16/46	10/42	0.405
BMI (kg/m^2^)	24.82 ± 4.45	23.04 ± 3.64	0.023∗
Smoking (yes/no)	29/33	3/49	<0.001∗∗
Pre-treatment HAMD-17	23.90 ± 4.60		
Post-treatment HAMD-17	8.43 ± 4.24		
Responsder	43/62		
Non-responder Incomplete data	7/62 12/62		

Sex and smoking, Chi-square test; age and BMI, Student's t-test; HAMD-17, Hamilton Depression Rating Scale, 17-item; SSRI, selective serotonin reuptake inhibitor; SNRI, serotonin–norepinephrine reuptake inhibitors.

### 3.2 Altered mRNA expression level of the transcription factors of TNIP1 in monocyte compared pretreatment MDD patients with health control

**Fig. 1:**
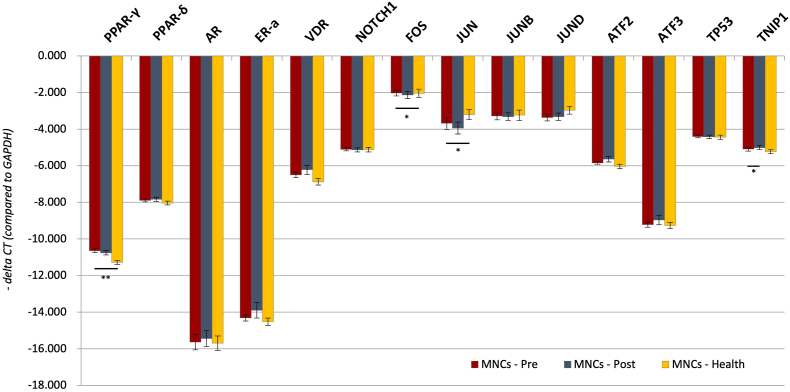
**mRNA expression level of TNIP1 transcription factors in monocytes of health control, pre-treatment and post-treatment group.** Post-treatment vs. pre-treatment: paired *t*-test; Health control vs. pre-treatment: ANCOVA, with adjustment of age, sex, BMI, and smoking. ∗*p* < 0.05, ∗∗*p* < 0.001.

To investigate the regulation of TNIP1 in MDD patients, we analyzed the mRNA expression levels of TNIP1 and its transcription factors in monocytes using ANCOVA, adjusted for age, gender, BMI, and smoking. Compared to healthy controls, pre-treatment MDD patients exhibited significantly higher expression levels of PPAR-γ (F = 6.213, *p* < 0.001) and FOS (F = 2.775, *p* = 0.023) and lower expression levels of JUN (F = 2.631, *p* = 0.029). No significant differences were observed for TNIP1 (F = 1.023, *p* = 0.408), PPAR-δ (F = 0.935, *p* = 0.462), AR (F = 0.781, *p* = 0.566), ERa (F = 1.274, *p* = 0.282), VDR (F = 1.521, *p* = 0.192), NOTCH1 (F = 1.424, *p* = 0.224), JUNB (F = 1.290, *p* = 0.276), JUND (F = 1.611, *p* = 0.166), ATF2 (F = 1.029, *p* = 0.406), ATF3 (F = 1.636, *p* = 0.159), or TP53 (F = 0.435, *p* = 0.823) (Table 2, Fig. 1).

**Table 2:** The transcript levels by -delta Ct value (standard deviation) of TNIP1 and its transcription factors: comparison between healthy controls and major depressive disorder patients before and after treatment.

	I MDD pre-treatment (n = 62)	II MDD post-treatment (n = 50)	III Healthy controls (n = 52)	I vs. III F- and *p*-value	I vs. II *p*-value
TNIP1	−5.09 (0.88)	−5.01 (0.80)	−5.23 (0.78)	F = 1.023 *p* = 0.408	*p* = 0.031∗
PPAR-γ	−10.64 (0.77)	−10.76 (0.87)	−11.28 (0.79)	F = 6.213 *p* < 0.001∗∗	*p* = 0.975
PPAR-δ	−7.89 (0.66)	−7.84 (0.80)	−8.04 (0.72)	F = 0.935 *p* = 0.462	*p* = 0.226
AR	−15.64 (3.01)	−15.44 (3.54)	−15.69 (2.72)	F = 0.781 *p* = 0.566	*p* = 0.474
ERa	−14.31 (1.24)	−13.90 (2.92)	−14.52 (1.31)	F = 1.274 *p* = 0.282	*p* = 0.829
VDR	−6.50 (1.00)	−6.22 (1.70)	−6.88 (1.13)	F = 1.521 *p* = 0.192	*p* = 0.130
NOTCH1	−5.10 (0.66)	−5.13 (0.73)	−5.12 (0.76)	F = 1.424 *p* = 0.224	*p* = 0.369
FOS	−2.03 (1.13)	−2.14 (1.30)	−2.04 (1.40)	F = 2.775 *p* = 0.023∗	*p* = 0.527
JUN	−3.68 (2.35)	−3.94 (2.19)	−3.20 (1.79)	F = 2.631 *p* = 0.029∗	*p* = 0.121
JUNB	−3.28 (1.51)	−3.32 (1.39)	−3.24 (1.48)	F = 1.290 *p* = 0.276	*p* = 0.386
JUND	−3.36 (1.40)	−3.32 (1.38)	−2.96 (1.35)	F = 1.611 *p* = 0.166	*p* = 0.920
ATF2	−5.84 (0.55)	−5.64 (1.08)	−6.04 (0.76)	F = 1.029 *p* = 0.406	*p* = 0.389
ATF3	−9.22 (0.98)	−8.97 (1.73)	−9.26 (1.11)	F = 1.636 *p* = 0.159	*p* = 0.479
TP53	−4.40 (0.51)	−4.42 (0.60)	−4.44 (0.74)	F = 0.435 *p* = 0.823	*p* = 0.973

I vs. III ANCOVA, with adjustment of age, sex, BMI, and smoking; I vs. II: paired *t*-test.

### 3.3 Elevated mRNA expression level of TNIP1 in monocyte in MDD patients after treatment

To assess changes in the TNIP1 pathway following treatment, we conducted paired t-tests comparing pre- and post-treatment MDD patients. The expression level of TNIP1 significantly increased in the post-treatment MDD group compared to the pre-treatment group (*p* = 0.031). However, no significant differences were observed for the transcription factors, including PPAR-γ (*p* = 0.975), PPAR-δ (*p* = 0.226), AR (*p* = 0.474), ERa (*p* = 0.829), VDR (*p* = 0.130), NOTCH1 (*p* = 0.369), FOS (*p* = 0.527), JUN (*p* = 0.121), JUNB (*p* = 0.386), JUND (*p* = 0.920), ATF2 (*p* = 0.389), ATF3 (*p* = 0.479), or TP53 (*p* = 0.973) (Table 2, Fig. 1).

### 3.4 A lower pretreatment PPAR-γ mRNA expression level in monocytes can predict better clinical efficacy

**Fig. 2:**
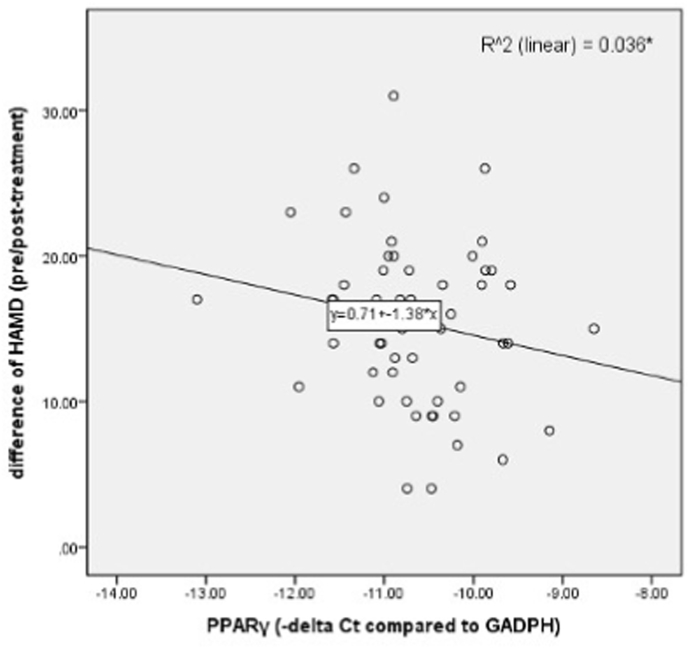
Linear regression of PPARγ level to difference of pre-treatment between post-treatment HAMD.

**Fig. 3:**
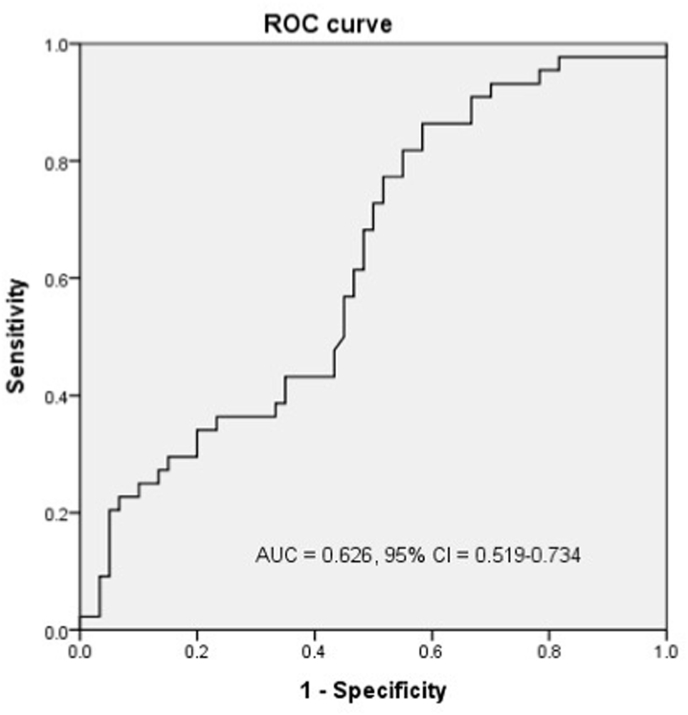
ROC analysis of mRNA expression level of PPARγ to stratify responder to non-responder.

To clarify the correlation between clinical improvement and the expression levels of TNIP1 transcription factors, we performed multiple linear regression analysis using pre-treatment expression levels and confounding factors (age, gender, BMI, smoking) to predict changes in HAMD-17 scores (pre-treatment minus post-treatment). Lower pre-treatment PPAR-γ expression levels were significantly associated with greater improvement in HAMD-17 scores (standardized coefficient = −0.417, *p* = 0.016). No significant associations were found for TNIP1 or other transcription factors (Table 3, Fig. 2). In addition, ROC analysis demonstrated that baseline PPAR-γ levels could effectively distinguish between responders and non-responders (Fig. 3).

**Table 3:** Multiple linear regression analysis for the association between HAMD-17 score difference (pre-treatment – post-treatment) and expression level (-delta Ct value) of TNIP1 and its transcription factors.

Independent factor	-delta HAMD-17		
	Standardized coefficient	t	*p*-value
TNIP1	−0.183	−1.110	0.276
PPAR-γ	−0.417	−2.551	0.016∗
PPAR-δ	−0.104	−0.624	0.759
AR	−0.008	−0.051	0.960
ERa	−0.126	−0.788	0.437
VDR	−0.072	−0.445	0.659
Notch1	0.132	0.823	0.416
FOS	0.003	0.020	0.984
JUN	−0.008	−0.046	0.964
JUNB	−0.071	−0.444	0.660
JUND	−0.017	−0.108	0.915
ATF2	0.089	0.550	0.586
ATF3	0.095	0.593	0.557
TP53	−0.052	−0.320	0.751
Gender	0.164	1.033	0.309
Age	−0.118	−0.715	0.480
Smoking	−0.314	−1.936	0.061
BMI	−0.084	0.523	0.604

∗*p* < 0.05.

## 4 Discussion

This study aims to investigate the role of the TNIP1 and its transcription factors in MDD. Compared to the healthy control group, the mRNA expression levels of TNIP1 transcription factors in monocytes of the MDD population showed significantly higher levels of PPAR-γ and FOS, while JUN exhibited significantly lower levels. When comparing the expression levels of TNIP1 transcription factors before and after treatment, TNIP1 was found to significantly increase following treatment; however, none of the transcription factors displayed significant changes. Furthermore, the correlation between the pre-treatment expression levels of TNIP1 and its transcription factors with the degree of depression improvement indicated that lower pre-treatment levels of PPAR-γ were associated with better therapeutic outcomes in alleviating depression. Previous data have shown that TNIP1 mRNA expression increases in monocytes of depressed patients after treatment (Hung et al., 2025). However, investigations into the transcriptional regulation of TNIP1 have been limited to a few studies using cell line assays or animal models (Chen et al., 2015; Ramirez et al., 2015). Notably, this is the first data demonstrating the mRNA levels of PPAR-γ, FOS, and JUN and their association with clinical response in depression population.

Our research indicates that MDD links to inflammatory responses, with transcription factors such as FOS, JUN (which form the AP-1 complex), and PPAR-γ pathway playing critical roles. In vitro studies have shown that the AP-1 complex can suppress the transcription of pro-inflammatory genes (Hsu et al., 1994), while PPAR-γ exerts anti-inflammatory effects. Our findings reveal that, compared to healthy controls, monocytes from MDD patients exhibit significantly higher expression of PPAR-γ and FOS, and lower expression of JUN. This pattern may reflect the regulatory dynamics where FOS promotes and JUN inhibits PPAR-γ expression (Hasenfuss et al., 2014). Additionallly, the stress-activated protein kinase known as JNK (c-JUN N-terminal kinase) may induce inflammation and stress responses through the phosphorylation of proteins associated with the glucocorticoid receptor, potentially leading to depressive disorders (Jovicic et al., 2015). In differential regulation analysis (DRA) to compare the transcriptomic profiles of peripheral blood lymphocytes from patients with MDD and subsyndromal symptomatic depression, JUN was found to be one of differentially regulated genes (Xu et al., 2015). In studies focused on depressive disorders, research targeting the JNK pathway has indicated that higher levels of downstream product phosphorylation are associated with neuroticism and a tendency for negative interpretations of past events (Simic et al., 2013). These results suggest that these molecules are involved in the inflammatory mechanisms underlying MDD.

In previous clinical trials, the effects of PPAR-γ on depression treatment has been clarified (Colle et al., 2017), demonstrating that PPAR-γ agonists can enhance treatment outcomes. Activation of PPAR-γ inhibits inflammatory responses by suppressing NF-κB activation, which in turn reduces the production of TNF-α (Wang et al., 2013). A key finding from this study is that lower baseline PPAR-γ expression can predict better therapeutic outcomes in the future. One possible explanation is that individuals with lower baseline PPAR-γ expression have greater potential to enhance their innate immune system's anti-inflammatory response, resulting in more significant improvement following treatment. In contrast, higher baseline PPAR-γ expression may indicate that an individual's anti-inflammatory mechanisms are already active in response to stress. However, these individuals still exhibit other psychopathological symptoms of MDD, suggesting that the anti-inflammatory mechanisms alone are insufficient to reverse the adverse effects of stress, leading to poorer expected therapeutic efficacy. An alternative explanation is that PPAR-γ levels may distinguish distinct subgroups of MDD patients, with only certain groups responding to traditional antidepressant treatments by increasing PPAR-γ expression (Hung et al., 2025).

In animal models, overexpression of TNIP1 has been shown to alleviate depressive-like behaviors, and in humans, higher TNIP1 expression in monocytes correlates positively with remission status. Under duloxetine treatment, TNIP1 modulates inflammatory responses through regulation of PPAR-γ (Hung et al., 2025). Consistently, pioglitazone, a PPAR-γ agonist, has been reported to reduce depressive symptoms in MDD patients with metabolic syndrome, particularly among those with elevated CRP levels (Kemp et al., 2014), and to improve affective and motivational deficits when added to standard therapy in bipolar depression (Sepanjnia et al., 2012). The antidepressant effects of PPAR-γ agonists are thought to involve suppression of NF-κB and AP-1 (FOS/JUN) signaling, thereby attenuating pro-inflammatory mediators such as IL-6 and TNF-α (Zolezzi et al., 2017). In diabetic models, PPAR-γ agonists also enhance hippocampal BDNF expression, contributing to improved cognitive and emotional outcomes (Kariharan et al., 2015). Taken together, these observations suggest that antidepressant-induced regulation of TNIP1 in MDD may arise from the concerted activity of multiple transcription factors, including PPAR-γ, NF-κB, and AP-1, rather than from the isolated action of a few. Such multifactorial regulation could represent a key molecular interface linking antidepressant treatment, immune modulation, and neuroplastic adaptation in depression. Further investigations are warranted to delineate how these interacting transcriptional networks collectively shape TNIP1 dynamics and contribute to treatment responsiveness in MDD.

There were several limitations of this study including: (1) The sample size after grouping is too small and needs to be increased in order to conduct further analysis on the subgroup of response/remission or medications categories; (2) There are changes in TNIP1 before and after treatment; however, transcription factors show no changes pre- and post-treatment. Therefore, the potential interactions between transcription factors require further exploration; (3) The discrepancy between TNIP1 expression and its transcription factors may reflect a synergistic interaction among multiple regulators rather than the effect of specific ones. Additionally, mRNA expression levels do not always correspond to protein abundance or nuclear localization, which could further account for the gap observed in our data. Further research can provide clearer insights through the analysis of products at different stages.

## 5 Conclusion

The integration of our findings with prior evidence on the potential antidepressant effects of PPAR-γ agonists suggests that PPAR-γ expression levels may influence treatment responsiveness in MDD. It is possible that lower baseline PPAR-γ expression could be associated with a more favorable response to conventional antidepressant therapies, whereas patients with higher PPAR-γ expression or inflammation-related features might potentially benefit from PPAR-γ-targeted interventions. While these observations warrant further validation, they may provide a basis for exploring personalized treatment strategies in MDD, emphasizing the importance of identifying molecular subgroups that could guide more tailored therapeutic approaches.

## CRediT authorship contribution statement

**Yu-Wei Lin:** Writing – original draft. **Daw-Yang Hwang:** Methodology, Formal analysis. **Yi-Yung Hung:** Writing – review & editing, Resources, Project administration, Investigation, Funding acquisition, Formal analysis, Data curation, Conceptualization.

## Declaration of competing interest

The authors declare that they have no known competing financial interests or personal relationships that could have appeared to influence the work reported in this paper.

## Data Availability

Data will be made available on request.
